# Engineering preferentially-aligned nitrogen-vacancy centre ensembles in CVD grown diamond

**DOI:** 10.1038/s41598-019-42314-7

**Published:** 2019-04-08

**Authors:** Christian Osterkamp, Martin Mangold, Johannes Lang, Priyadharshini Balasubramanian, Tokuyuki Teraji, Boris Naydenov, Fedor Jelezko

**Affiliations:** 1 Institute for Quantum Optics and Center for Integrated Quantum Science and Technology (IQST), Ulm University, Albert Einstein Allee 11, Ulm, 89081 Germany; 2 Institute for Electron Devices and Circuits, Ulm University, Albert Einstein Allee 45, Ulm, 89081 Germany; 3 Wide Bandgap Materials Group, Research Center for Functional Materials, National Institute for Material Science, 1-1, Namiki, Tsukuba, Ibaraki, 305-0044 Japan; 4 Present Address: Helmholtz-Zentrum Berlin für Materialien und Energie (HZB), Kekuléstraße 5, Berlin, 12489 Germany

## Abstract

Here we report a method for improving the magnetic field sensitivity of an ensemble of Nitrogen-Vacancy (NV) centres in ^12^C-enriched diamond aligned along the [111] crystal axis. The preferentially-aligned NV centres are fabricated by a Plasma Enhanced Chemical Vapour Deposition (PECVD) process and their concentration is quantitatively determined by analysing the confocal microscopy images. We further observe that annealing the samples at high temperature (1500 °C) in vacuum leads to a conversion of substitutional nitrogen into NV centres. This treatment also increases the coherence time of the NV centres electron spins up to 40 μs, which corresponds to enhancement of the sensitivity by a factor of three. However, this procedure also leads to a loss of the preferential alignment by 34%.

## Introduction

The Nitrogen-Vacancy (NV) colour centre in diamond is one of the most promising physical systems in the emerging field of quantum technology due to its unique properties^1^. It has been demonstrated that NVs can serve as universal sensors for electric^2^ and magnetic fields^3,4^, temperature^5^ and pressure^6^ with high sensitivity and nano-scale spatial resolution. This is achieved since the electron spin associated with a single NV can be manipulated and readout at ambient conditions. For magnetic field sensing with an ensemble of *N* NV centres, the sensitivity is given by^7^,

1 $$\eta \approx \frac{h}{g\cdot {\mu }_{B}}\frac{1}{C\sqrt{N\cdot \tau }}$$

where *h* is the Planck constant, *g* the Landé factor, *μ*_*B*_ is the Bohr magneton and *C* the measurement contrast. The NV’s coherence time is represented by *τ*, set to T_2_ measured by a Hahn echo^8^ or ${T}_{2}^{\ast }=\frac{1}{\pi {\rm{\Delta }}\nu }$ with Δ*ν* being the NVs line width for ac and dc magnetometry, respectively. Most of the studies use single NVs, but in many cases an ensemble of NVs is required due to better sensitivity (as shown above) and due to the possibility to perform field imaging^9–11^ (imaging can be realized via scanning probe techniques^3^, but it is usually slower). For most of these studies, diamond crystals with a [100] crystal orientation of the surface have been used, where the NVs have four possible orientations. Often, a [111] orientation is preferred since in this case the optical dipole associated with the NV centres is parallel to the surface and thus it is the optimal orientation for the emission of fluorescence. Often, it is difficult to work with several orientations and preferentially-aligned NVs are required, as the number of collected photons increases and therefore the signal-to-noise ratio in sensing experiments.

In previous studies, it has been shown that single NVs can be aligned along two different orientations^12^, followed by the possibility to align up to 94% of single NV centres along the [111] crystal orientation^13–15^, when choosing a [111] oriented diamond substrate. In recent reports, an alignment ratio of an NV ensemble above 99% was demonstrated^16^ and also shallow ensembles approximately 10 nm away from the surface were created^17^. More recently the thermal stability of aligned NVs in thick layers (≈ 25 *μ*m) was studied^18^, where it was observed that the alignment can be broken and even the NV centres can be destroyed. All NV ensembles in these studies often show a relatively short Hahn echo coherence time going up to 6 *μ*s.

In the current work we achieve an order of magnitude longer T_2_ for ensembles of NV centres with comparable concentrations where we show that high temperature annealing of CVD grown diamond in vacuum leads to enhanced magnetic field sensitivity without loosing NV centres.

## Results

### Sample Analysis

In this work we report on engineering nitrogen doped isotopically enriched diamond layers for magnetometry applications with NV centres preferentially-aligned along the [111] crystal axis. We use purified ^12^CH_4_ gas (99.999%) for all processes, which already contains nitrogen with a concentration of approximately 70 ppm according to the supplier (Cambridge Isotope Laboratories). In the first experiment we fabricated a 40 nm thick diamond layer with a high concentration of NV centres using Plasma Enhanced Chemical Vapour Deposition (PECVD) on a IIa [111] diamond substrate (Element Six Group). Optically Detected Magnetic Resonance (ODMR) measurements revealed that all NVs were oriented along the [111] crystal axis, see Fig. 2(d). With this sample (referred to as Sample I) and applying the method described in^19^ we were able to detect hydrogen Nuclear Magnetic Resonance (NMR) signal coming from the immersion oil used in our confocal microscope and also to determine the average depth of the NVs to be 8 nm (see Supplementary Information). The coherence time measured via Hahn echo decay was found to be T_2_ = 4 *μ*s, but we expected a much longer value for the given ^12^C concentration as reported previously^20^. This value is even shorter for a diamond with natural abundance of ^13^C nuclear spins, where it is expected that T_2_ ≈ 500 *μ*s^21^. Thus we can conclude that T_2_ is limited by the presence of paramagnetic defects, probably nitrogen related ones such as substitutional nitrogen atoms (P1 centres), NVH and NVN (H3), but also other vacancy related defects (R5, R6 or R10)^22^.

**Figure 1:**
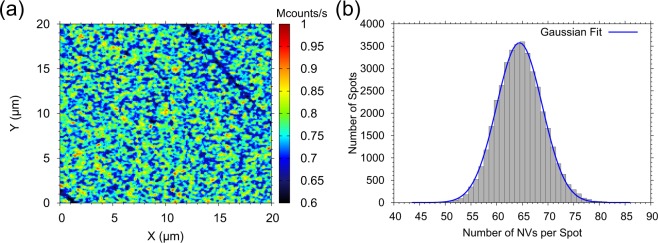
Confocal image from Sample A (**a**) and the resulting histogram (**b**), including the Gaussian fit (blue curve), after the analysis, see text for details.

In order to study the fabrication process in more detail and to improve the spin properties, we fabricated two additional samples at different conditions (diamond substrates type IIa from Applied Diamond Inc., with an intrinsic nitrogen concentration below 1 ppm). The layers were grown in our home-built PECVD reactor with different microwave powers which results in different plasma densities, growth temperatures and therefore different growth rates. The latter are important for tuning the nitrogen incorporation and thus the NV formation in the diamond. The parameters for the individual growth processes are summarized in Table 1. The grown layers are investigated with a home-built confocal microscope set-up where we observe a high NV concentration (>4.8 · 10^14^/cm^3^) in all samples. We have developed a method for analysing the confocal images to determine the concentration of NV centres on an area of 20 *μ*m × 20 *μ*m. First we take a confocal image as shown in Fig. 1(a), where NVs appear as bright spots. In a next step, a calibration of the fluorescence signal has to be performed, therefore we measure a single NV centre, confirmed by a second order auto correlation function *g*^(2)^ experiment^23^, and average the photon time trace, to receive the count rate of a single NV. The count rate of each pixel of the confocal image is then normalized with this value and since the spatial resolution of the fluorescence signal of our sample is diffraction limited, we obtain a count rate per confocal volume (defined simply as spot). The point spread function is distributed over several pixels. In a last step the number of NV centres per spot is calculated for the whole confocal image and is plotted as a histogram in Fig. 1(b). This distribution is fitted with a Gaussian function to obtain the averaged number of NV centres per confocal volume. The analysis method is repeated for several confocal images for better statistics. It is interesting to note that the standard deviation of the fit is nearly the square root of the mean value, indicating that the emitters (NV centres) in the sample follow a Poisson distribution.

**Table 1:** Summary of the parameters used for the PECVD process and the obtained results (*ν*: growth rate, *δ*: growth layer thickness).

Sample	MW Power, Watt	Temp., °C	*ν*, nm/hour	*δ*, nm	NV conc. as-grown, ppb	NV conc. anneal., ppb	T_2_ as-grown, *μ*s	T_2_ anneal., *μ*s
Sample I	1200	760*	20**	40**	640.0	—***	4	—***
Sample A	1200	930	210	420	10.3	12.5	10.1 ± 2.6	32.3 ± 4.2
Sample B	720	875	120	240	3.8	17.3	6 ± 3.3	26 ± 4.4

Conditions of the growth processes are: growth time t = 2 h, microwave (MW) frequency f = 2.46 GHz, pressure p = 22.5 mbar, methane concentration c_0_ = 0.5% for Samples A and B and c_*I*_ = 0.05% for Sample I with respect to hydrogen which was applied with a flow of 200 sccm.

*Temperature of Sample I was measured with a thermocouple whereas the others were measured via an infra-red pyrometer.

**Estimated from SIMS results of another sample with same growth conditions.

***No annealing procedure was performed on this sample.

### NV centre analysis and coherence property study

**Figure 2:**
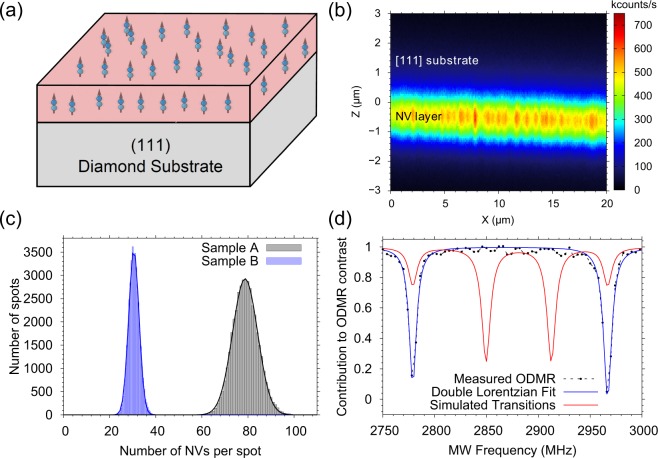
(**a**) Schematic drawing of the diamond layers fabricated in this work. The arrows represent NV centres aligned along the [111] crystal axis. (**b**) Confocal image showing a cross section of a sample, where the fluorescence from the NVs in the diamond layer is labelled. (**c**) Histogram from the analysis of Samples A and B, showing the increased number of NVs in the layer grown at higher MW power. (**d**) ODMR spectrum showing the preferential alignment of the ensemble of NV centres. Black points are experimentally measured data whereas the blue solid line is a Lorentzian Fit to the data and the red line represents the simulated ODMR transitions at the applied external magnetic field. The transitions at 2776 Mhz and 2964 Mhz belong to [111] oriented NVs whereas the other correspond to the other three directions.

The above described technique is used for both Samples (A and B) and we observe a good separation of the corresponding histograms, as shown in Fig. 2(c). The data shows that the NV density increases with microwave power, which is in contradiction with previous studies^16^. The temperatures during the growth processes are measured by a pyrometer focused on the diamond’s surface and listed in Table 1. Unfortunately we could not run the CVD process at a microwave power lower than 720 W since the plasma becomes unstable. It is also worth noting that our CVD system is equipped with a graphite heated stage which keeps the temperature at 750 °C even when the source of microwave plasma is completely switched off. ODMR measurements of all samples confirm the presence of NV^−^ centres and prove preferential alignment along the [111] orientation. In other words, the NV quantization axis (the line connecting the nitrogen atom and the vacancy) is perpendicular to the diamond surface, as illustrated in Fig. 2(a). These measurements are performed in a static magnetic field with the B-field vector pointing along the [111] direction. In this configuration, NV centres oriented along the other three directions make the same angle (≈ 70.5°) to the applied magnetic field, and hence the resonance frequencies overlap. Figure 2(d), shows the simulated and measured ODMR spectrum at approximately 33 G, exemplary taken from Sample A. The simulation shows the relative contribution of different NV orientations compared with maximum ODMR contrast (≈ 30%). The simulation is performed with the assumption that all four NV orientations are equally present in the sample, which is markedly different from the experimental observation. From the measured data, we estimate that about 99% of the NV centres have the same orientation.

**Figure 3:**
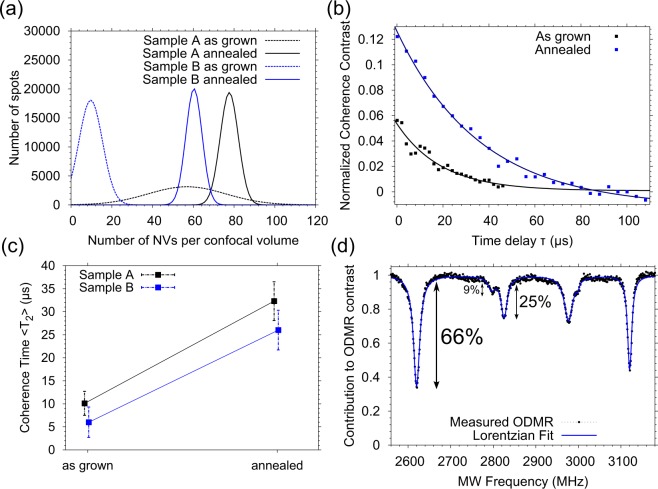
(**a**) Gaussian fitted number of NVs in a confocal spot and (**b**) Hahn echo decay of NV ensembles from Samples A and B before and after the annealing procedure. (**c**) Increase of the average Hahn echo coherence time T_2_ of both Samples A and B due to the annealing treatment. (**d**) ODMR spectrum showing the break of the preferential alignment. As in Fig. 2(d) the black points represent experimentally measured ODMR signal and the blue solid line is a Lorentzian Fit. Additional to the [111] oriented NVs (66% of all NVs) we observe NVs in other directions (34%).

We further investigated the spin properties of the NV ensembles, where we find that the average coherence time T_2_, of more than ten measured ensembles in Sample A, is about 10 *μ*s, see Table 1 (see also Supplementary Information for individual measurements). Again we find that T_2_ is limited by some other spin defects, probably in the form of P1 centres or electron traps. Motivated by previous reports^24,25^ on annealing of diamonds with NV centres at high temperatures, we decided to perform similar treatment here. Both samples were annealed at a temperature of 1500 °C in ultra high vacuum (below 10^−7^ mbar) for two hours. Under these conditions vacancies and vacancy related defects as described above, created during the crystal growth, become mobile and start to diffuse. Some of them are destroyed at the surface, but a certain amount is captured by nitrogen atoms and form additional NV centres. This effect can be seen in the analysis data shown in Fig. 3(a), where the Gaussian fit of the histograms after the annealing is shifted to higher NV numbers. The increase for Sample A (high MW power) is from 66 to 81 NVs per confocal volume whereas the increase for Sample B (low MW power) is from 14 to 64 NVs per confocal volume. Furthermore, we observe an increase of the Hahn echo coherence time T_2_ as shown in Fig. 3(c). For the as-grown NVs in Sample A, we measured an average Hahn echo coherence time of 10 *μ*s, while after the annealing procedure we find T_2_ values up to 40 *μ*s (detailed overview of Hahn Echo measurements for different ensembles are shown in the Supplementary Information). This effect has been reported previously as mentioned above and it is believed that at those temperatures various paramagnetic defects are destroyed, thus leading to an increase of NVs’ T_2_. In the ODMR spectra of the NV centres after the annealing treatment from Sample A, shown in Fig. 3(d), two additional spectral lines are found (two lines instead of one are found, as the external magnetic field is not perfectly aligned along the [111] crystal axis but with a slight off angle of approximately 3 degrees). This observation is a result of breaking the preferential alignment or in other words, there are NV centres which are not aligned along the [111] crystal axis. The proposed mechanism for diffusion of NV centres at high temperatures^26^ can explain this effect. Another explanation would be that during the annealing new NV centres are formed, which are randomly aligned along the four possible orientations. By analysing the intensity of the lines in Fig. 3(d), we find that only 66% of the NVs are preferentially-aligned along the [111] crystal axis and 34% have other orientations, which decreases the ODMR contrast and therefore the magnetic field sensitivity by the same value.

In Table 2 we compare the sensitivity of ensembles created by different methods, keeping in mind that equation (1) has to be interpreted carefully as a larger detector size does not automatically translate into better sensitivity. Depending on the application, the number of NV sensors has to be optimized as for example in the case of magnetometry applications, NV centres in equal distance to the signal source are required, as the signal decays strongly with distance. Hence, a better benchmark for evaluating the quality of a sample is to give a sensitivity per root volume and therefore we modify equation (1), for a pulsed measurement scheme to a volume normalised sensitivity *η*_*V*_:

2 $${\eta }_{V}\approx \frac{h}{g\cdot {\mu }_{B}}\frac{1}{{C}_{O}\cdot \sqrt{{n}_{B}\cdot {p}_{B}\cdot {\tau }_{L}}}\frac{1}{\sqrt{{T}_{2}}}$$

where *C*_*O*_ is the measurement contrast, *n*_*B*_ = *N*/*V* the sensor density (with N: number of NVs, V: volume), *p*_*B*_ the average rate of detected photons and *τ*_*L*_ the duration of the readout laser pulse^7^. The estimated sensitivity of our samples are similar to previously reported values for preferentially aligned CVD grown NVs (calculated from^17^). In comparison to ion implantation technique^9^ the sensitivities of CVD grown NVs are better, as it is not possible to produce preferentially aligned NVs by ion bombardment. The best values reported so far are achieved with electron irradiated [111] oriented diamond fabricated via High Pressure High Temperature method (HPHT)^27^ where an expected sensitivity of 100 ${\rm{f}}\,{\rm{T}}/\sqrt{{\rm{H}}{\rm{z}}}$ using a pulsed detection scheme with discrete readout steps is used. Independently of the overall sensitivity in our samples, the improvement factors resulting from our vacuum annealing treatment are 1.3 and 2.9, respectively for Samples A and B.

**Table 2:** Summary of the calculated AC volume normalised magnetic field sensitivities reached in Samples A and B as-grown and after the high temperature vacuum annealing procedure and in Sample I as-grown as well as the values for an earlier CVD overgrowth experiment, *calculated from data given in^17^ and an electron irradiated HPHT diamond. (Parameters *C*_*O*_ = 0.2, *p*_*B*_ = 100 kcts/s and *τ*_*L*_ = 300 ns were used).

Volume normalised sensitivity *η*_*V*_ in $\frac{{\bf{1}}{{\bf{0}}}^{{\boldsymbol{-}}7}{\bf{n}}{\bf{T}}}{\sqrt{{\bf{H}}{\bf{z}}}\sqrt{{\bf{c}}{{\bf{m}}}^{{\bf{3}}}}}$						
Sample A		Sample B		Sample I	CVD grown*	HPHT sample^27^
as-grown	annealed	as-grown	annealed	as-grown	as-grown	e-irradiated
9.6	7.3	20.4	6.9	0.4	5.8	8.3

### Secondary Ion Mass Spectroscopy (SIMS)

**Figure 4:**
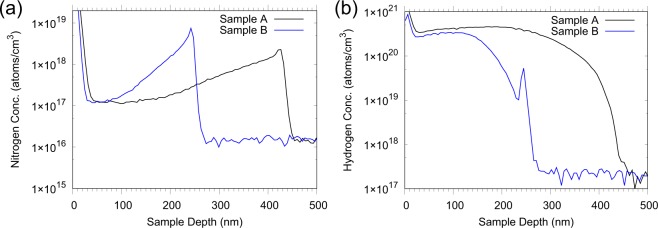
SIMS measurement showing the distribution of nitrogen (**a**) and hydrogen (**b**) atoms within the layers in Samples A and B.

In order to gain more information about the composition of the diamond layers we performed secondary ion mass spectrometry (SIMS) measurements, shown in Fig. 4. The first important information from the SIMS data is that the growth rates for both processes are different. The growth time was set to two hours in both cases and the calculated growth rates are given in Table 1. Another interesting result is the varying nitrogen density within the layer, see Fig. 4(a). At the beginning of the growth process there is a maximum of the nitrogen concentration and then it decays monotonically towards the surface. Since no nitrogen gas was introduced in the chamber, it could be concluded that there is some limited nitrogen source, which is used up during the growth. Another possible source of nitrogen would be contamination of the methane gas, however this would mean a constant nitrogen concentration within the diamond layers. The total amount of nitrogen in the layers, after the annealing processes, was determined to be 1.07 × 10^12^ and 1.02 × 10^12^ atoms, from where we calculated the conversion efficiency from nitrogen to NV centres to be 0.25% and 0.21%, for Samples A and B respectively. These results are in good agreement to previous studies where the NV^−^ concentration with respect to the total nitrogen content is approximately 1:300 (conversion efficiency 0.33%)^12^. The hydrogen SIMS data (Fig. 4(b)) show about three orders of magnitude higher hydrogen concentration in the layer compared to the substrate. From the data we calculate that the concentration varies within the layer from 10 to 3000 ppm, values which are still puzzling for us, though in earlier reports similar concentrations have been reported for polycrystalline CVD layers^28,29^. The hydrogen is either bond only to carbon to form the H1^29,30^ and H2^29^ defect centres or it is bound to an NV building the NVH^31^ centre. All these defects have been also observed in mono-crystalline diamond layers^30,31^, they are paramagnetic and stable at temperatures similar to our treatment here^28,30^. The high concentration of these spin impurities could explain the short values of the coherence time of the NVs compared to the case where the limiting factor is the presence of ^13^C nuclear spins.

## Conclusion

In conclusion we have shown that the creation efficiency of NV ensembles in [111] oriented CVD grown diamond layers can be improved by high temperature annealing in vacuum. Moreover, this treatment leads to an increase of the coherence time as well as to a loss of the preferential alignment, where by using equation (2), this results in an enhancement of the magnetic field sensitivity by a factor of 2.9. However, the coherence times in those samples are shorter than the one expected for a limit posed by the dynamics of the ^13^C nuclear spin bath. This limitation is due to paramagnetic defects present in the grown layer, where possible candidates could be H1 and H2 centres suggested by the high hydrogen concentration revealed by the SIMS measurements.

## Methods

### CVD Diamond growth process

The diamond layers were produced in a home-built microwave assisted chemical vapour deposition chamber which consists of an external cavity (the vacuum chamber itself), a conical microwave coupling structure, a heated substrate holder and an inner cavity for plasma creation^32^. The microwave generator (Muegge electronic, type MW-GPEOM77B-5K-04\85) operates at an output frequency of 2.46 GHz and an output power up to 6 kW. The substrate holder is equipped with a graphite heater running at 1.5 kW which leads to a diamond substrate temperature of approximately 750 °C, measured with an infra-red pyrometer (Optris, type CTlaser 1 M\2 M) which operates at a wavelength of 1.6 *μ*m. All gases used in this study are additionally purified. The hydrogen (chemical purity 99.99999%) by a palladium filter (Johnson Matthey, type Hydrogen Purifier HP-25) and the isotopically purified methane (isotopical purity 99.999%) by a heated getter device (MonoTorr, type PS4-MT3-531).

### Confocal microscopy analysis

All experiments have been performed on a home built confocal microscope set-up equipped with a 515 nm pulsed diode laser (Toptica Photonics, type iBeam-smart-515-S). The electron spin manipulation was realised using a continuous wave MW source (Rohde und Schwarz, type SMIQ04B), a MW switch (Mini circuits, type ZASWA-2-50DR+) and an arbitrary waveform generator to form MW pulses (Tektronix, AWG7122C). The constant magnetic field has been provided by a permanent magnet which was mounted on a rotational 3D stage (Thorlabs, type PT03 and CR01). The experiments were controlled with the qudi software package^33^.

### Annealing process

The diamond samples were annealed in a high vacuum chamber (<10^−7^ mbar), equipped with a boron-nitride heater (Tectra, type boralectric heater HTR-1001) with a maximum temperature of 1500 °C.

## Supplementary information

Supplementary Information
