# Supplementary material for: Engineering preferentially-aligned nitrogen-vacancy centre ensembles in CVD grown diamond
Source: Sci Rep. 2019 Apr 8;9:5786. doi: 10.1038/s41598-019-42314-7 (PMC6453974; doi:10.1038/s41598-019-42314-7)
Supplement: Supplementary file 1 — Supplementary Information [file 41598_2019_42314_MOESM1_ESM.pdf]

Supplementary Information on Engineering preferentially-aligned nitrogen-vacancy centre ensembles in CVD grown diamond

Christian Osterkamp[1,2,*], Martin Mangold[1], Johannes Lang[1], Priyadharshini Balasubramanian[1], Tokuyuki Teraji[3], Boris Naydenov[1,a], and Fedor Jelezko[1]

[1]Institute for Quantum Optics and Center for Integrated Quantum Science and Technology (IQST), Ulm University, Albert Einstein Allee 11, Ulm 89081, Germany
[2]Institute for Electron Devices and Circuits, Ulm University, Albert Einstein Allee 45, Ulm 89081, Germany
[3]Wide Bandgap Materials Group, Research Center for Functional Materials, National Institute for Material Science, 1-1, Namiki, Tsukuba, Ibaraki 305-0044 Japan
[*]E-mail:christian.osterkamp@uni-ulm.de
[a]Present address: Helmholtz-Zentrum Berlin für Materialien und Energie (HZB) , Kekuléstraße 5, Berlin 12489, Germany

1 Nuclear Magnetic Resonance signal of Hydrogen spins on the diamond surface

The detection of hydrogen spins from the immersion oil on top of the diamond surface has become a state-of-the-art technique to determine the depth of NV centres. The XY8 protocol as described in[1–3] is applied at a static magnetic field of 637G, which is measured by the resonance shift of the NV centres electron spins.The resulting spectrum represents the convolution of the magnetic field caused by proton spins with the filter function defined by the XY8 pulse sequence. In order to extract the depth of the NV centres, the measured signal has to be deconvoluted and fitted with a Lorentzian as can be seen in figure 1. This figure also shows the expected value for the Larmor frequency of the hydrogen spins (here 2.712 MHz) as derived from the magnetic field mentioned above. From the area under the peak in figure 1 and following the analysis described in[3] the depth of the NV centres can be determined to be 8 nm.

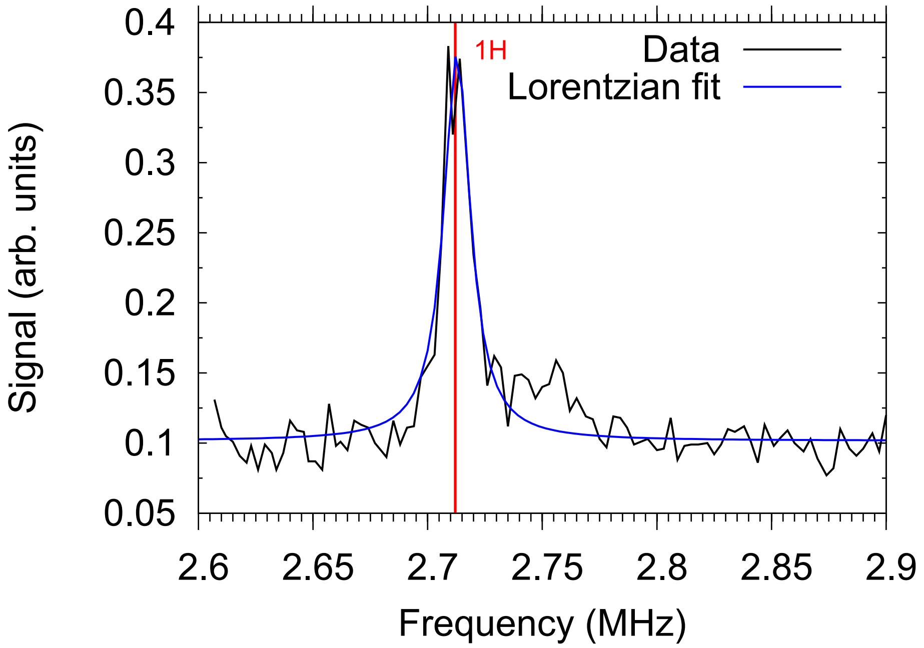

Figure 1. Proton NMR spectrum of immersion oil on top of the diamond's surface. The applied external magnetic field is 637 G, showing a resonance line at 2.712 MHz. Analysing the signal reveals a depth of the NV centres of about 8 nm.

2 Hahn Echo measurements on Samples A and B

Coherence times in our work are measured at a magnetic field of around 50 Gauss with the Hahn echo sequence and the underlying data for the average values given in the main part figure 3 and table 1 are shown in the following table 1.

	Sample A, as-grown	Sample A, annealed	Sample B, as-grown	Sample B, annealed
Ensemble 01	7.9 μs	29.4 μs	8.0 μs	24.0 μs
Ensemble 02	11.4 μs	31.0 μs	13.0 μs	22.0 μs
Ensemble 03	9.4 μs	40.0 μs	4.4 μs	30.0 μs
Ensemble 04	11.0 μs	29.0 μs	8.5 μs	24.5 μs
Ensemble 05	15.0 μs	33.0 μs	5.3 μs	22.6 μs
Ensemble 06	10.0 μs	38.0 μs	3.1 μs	21.5 μs
Ensemble 07	12.5 μs	28.3 μs	2.8 μs	35.0 μs
Ensemble 08	11.4 μs	29.0 μs	5.2 μs	33.0 μs
Ensemble 09	9.6 μs	33.0 μs	3.4 μs	23.1 μs
Ensemble 10	10.2 μs	-	-	22.0 μs
Ensemble 11	9.5 μs	-	-	24.0 μs
Ensemble 12	9.1 μs	-	-	26.8 μs
Ensemble 13	2.8 μs	-	-	29.0 μs
Ensemble 14	10.3 μs	-	-	-
Ensemble 15	11.7 μs	-	-	-
**Average**	**10.1 μs**	**32.3 μs**	**6.0 μs**	**26.0 μs**

Table 1. Summary of the coherence times of Samples A and B which are used to determine the average values given in the table 1 and figure 3.

References

1. Staudacher, T. *et al.* Nuclear magnetic resonance spectroscopy on a (5-nanometer)3 sample volume. *Science* **339**, 561–563, DOI: 10.1126/science.1231675 (2013). http://science.sciencemag.org/content/339/6119/561.full.pdf.

2. Müller, C. *et al.* Nuclear magnetic resonance spectroscopy with single spin sensitivity. *Nat. Commun.* **5**, 4703, DOI: 10.1038/ncomms5703 (2014).

3. Pham, L. M. *et al.* Nmr technique for determining the depth of shallow nitrogen-vacancy centers in diamond. *Phys. Rev. B* **93**, 045425, DOI: 10.1103/PhysRevB.93.045425 (2016).
